# Pleistocene to holocene expansion of the black-belt cichlid in Central America, *Vieja maculicauda* (Teleostei: Cichlidae)

**DOI:** 10.1371/journal.pone.0178439

**Published:** 2017-05-30

**Authors:** Caleb D. McMahan, Luke Ginger, Marcy Cage, Kyle T. David, Prosanta Chakrabarty, Mark Johnston, Wilfredo A. Matamoros

**Affiliations:** 1 The Field Museum of Natural History, Chicago, Illinois, United States of America; 2 LSU Museum of Natural Science, Department of Biological Sciences, Louisiana State University, Baton Rouge, Louisiana, United States of America; 3 Universidad de Ciencias y Artes de Chiapas, Instituto de Ciencias Biológicas, Museo de Zoología, Tuxtla Gutierrez, Chiapas, Mexico; National Cheng Kung University, TAIWAN

## Abstract

The distributions of many Northern Hemisphere organisms have been influenced by fluctuations in sea level and climatic conditions during Pleistocene interglacial periods. These cycles are associated with range contraction and refugia for northern-distributed organisms as a response to glaciers. However, lower sea levels in the tropics and sub-tropics created available habitat for expansion of the ranges of freshwater organisms. The goal of this study was to use ecological niche modeling to test the hypothesis of north to south range expansion of *Vieja maculicauda* associated with Pleistocene glacial cycles. Understanding the biogeography of this widespread species may help us better understand the geology and interconnectivity of Central American freshwaters. Occurrence data for *V*. *maculicauda* was based on georeferencing of all museum records of specimens recovered from FishNet2. General patterns of phylogeographic structure were assessed with mtDNA. Present day niche models were generated and subsequently projected onto paleoclimatic maps of the region during the Last Interglacial, Last Glacial Maximum, and mid-Holocene. Phylogenetic analysis of mtDNA sequence data showed no phylogeographic structure throughout the range of this widespread species. Present day niche models were congruent with the observed distribution of *V*. *maculicauda* in Central America. Results showed a lack of suitable freshwater habitat in northern Central America and Mexico during the Last Interglacial, with greatest range expansion during the Last Glacial Maximum and mid-Holocene. Results support the hypothesis of a north to south range expansion of *V*. *maculicauda* associated with glacial cycles. The wide distribution of this species compared to other closely related cichlids indicates the latter did not respond to the degree of *V*. *maculicauda* in expansion of their distributions. Future work aimed at comparisons with other species and modeling of future climatic scenarios will be a fruitful area of investigation.

## Introduction

Understanding the contribution of historical and ecological processes that have shaped species distributions is a central goal of biogeography [1]. Species’ distributional patterns are dependent on a combination of factors related to the evolutionary history of a lineage and the geological events that have shaped its habitat, as well as the influence of other abiotic (e.g. climate, habitat availability) and biotic factors (e.g. competition, predation).

The distributions of many Northern Hemisphere organisms have likely been influenced by fluctuations in sea level and climatic conditions during Pleistocene interglacial periods [2–3]. Many studies illustrate a biogeographic history of range contraction followed by expansion during this time: with a contraction during the cooler and drier Last Glacial Maximum (LGM) and subsequent expansion during the warmer, wetter Holocene [4–5]. This pattern has been shown across a wide range of taxa, including freshwater fishes [2]. While the LGM is often associated with range contraction and refugia for many northern-distributed organisms as a response to glaciers, lower sea levels in the tropics and sub-tropics likely created available habitat for expansion of the ranges of some taxa, particularly freshwater organisms [6].

Central America hosts a diverse array of freshwater fishes that have been the basis of numerous evolutionary, ecological, and biogeographic studies [7–11]. These studies have uncovered interesting biogeographic patterns attributed to the complex geological history, physiography, and topography of Central America and surrounding areas [11–13].

**Fig 1:**
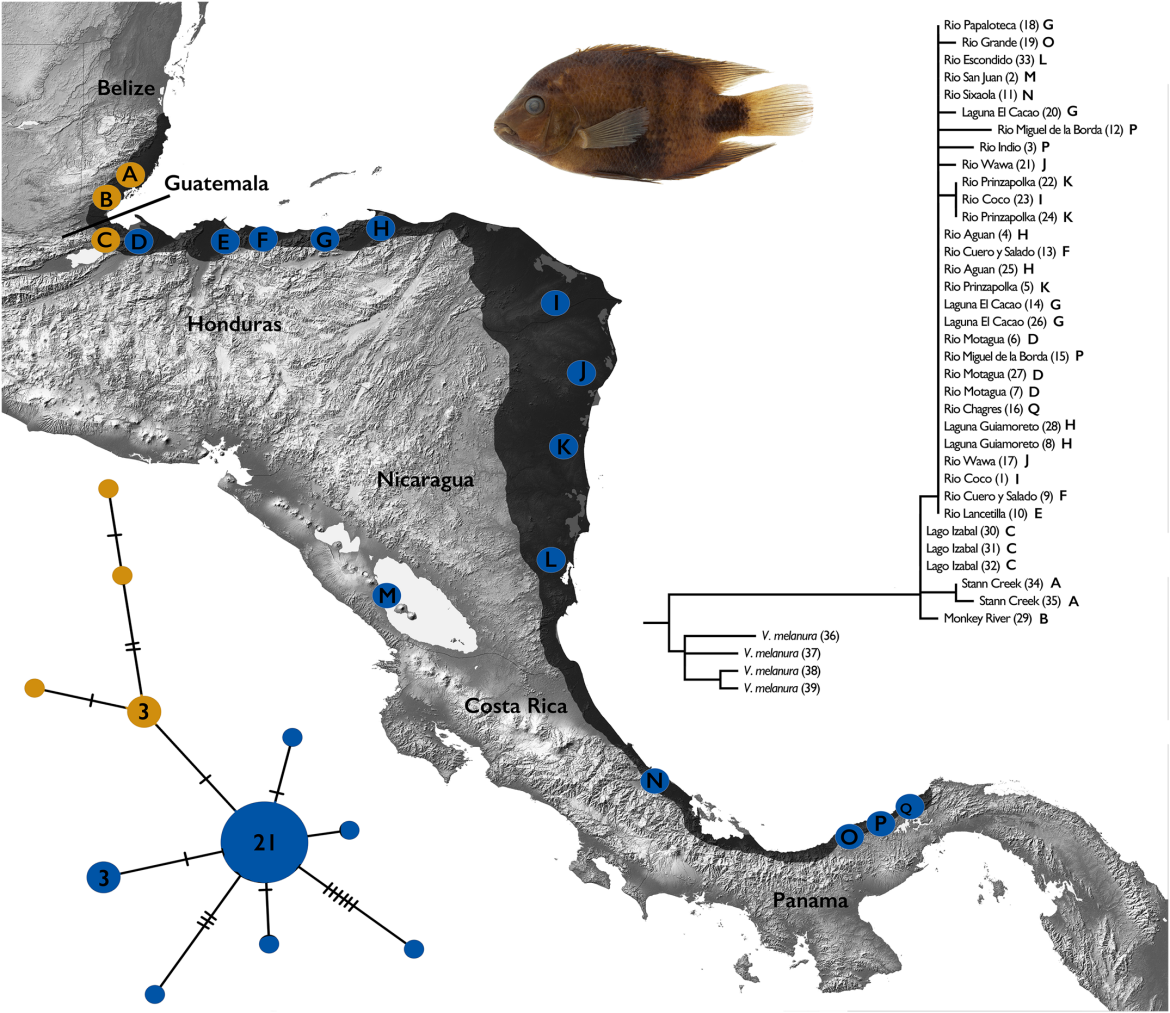
Distribution, sampling localities, and phylogeographic results for *Vieja maculicauda*. Distribution of *V*. *maculicauda* shown in shaded area on map, including Lago Nicaragua. Dots on map represent sampling localities for specimens used in phylogenetic and haplotype analyses. Numbers beside localities on the Bayesian phylogenetic tree correspond to list of samples in Table 1; letters correspond to localities on map. Numbers in haplotype network indicate number of individuals with that haplotype; circles without numbers equal one individual. Haplotype colors correspond to geographic patterns north and south of the area of the Motagua Fault.

The Black-belt cichlid, *Vieja maculicauda* [14], is somewhat unique in being widely distributed across lowland reaches of rivers in the Atlantic slope of Central America (Fig 1). While possessing a sizeable distribution, this notable species is largely restricted to the lowland portions of rivers from Belize south to the Río Chagres drainage in Panama [11]. The widespread distribution of this species stimulates interesting questions regarding biogeographic history. The distribution of *V*. *maculicauda* is particularly interesting as it crosses over multiple geologic breaks in Central America (Mayan Block, Chortís Block, Costa Rica-Panama Arc, and the Isthmus of Panama), which are shown to have played key roles in shaping the evolution of biota in the region [8–9,11,15–16]. However, the widespread distribution of this cichlid should not be confused with abundance and commonality, as this species is uncommon in various portions of its range [17]. *Vieja maculicauda* belongs to a clade of cichlids hypothesized to have originated within the region of Northern Middle America, associated with the vast Río Grijalva-Usumacinta drainage basin [11,18–19].

The first objective of this study was to assess mitochondrial patterns of phylogeographic structure within the widespread cichlid *V*. *maculicauda*. We then used ecological niche modeling methodologies, associated with contemporary and historical climates, to make inferences regarding the biogeographic history of this freshwater fish. These models and methods allowed us to test the hypothesis that northern to southern dispersal of this species was associated with the increase in available habitat during the LGM. Understanding the biogeography of this widespread species may help us better understand the geology and interconnectivity of Central American freshwaters.

## Materials and methods

### Specimen collection

Specimens of *V*. *maculicauda* were collected from throughout the distribution of the species (Belize, south to Panama) using seines and cast nets. Fishes were then euthanized with an overdose of MS-222 prior to preservation. Tissue samples (muscle and/or fin clips) were preserved in 95% ethanol. Voucher specimens were subsequently preserved with 10% formalin, stored in 70% ethanol, and deposited in the Collection of Fishes at the LSU Museum of Natural Science (LSUMZ). Museum collections supplied additional samples to fill in gaps, generating the best distributional sampling possible at present. The widespread distribution of this species is not equivalent with being common or abundant in all parts of its range. A detailed listing of samples and localities is reported in Table 1. A total of 35 individuals of *V*. *maculicauda* were sequenced and included in analyses.

**Table 1:** Tissue samples of *V*. *maculicauda* used for molecular phylogenetic analyses. Museum acronyms are as follows: LSUMZ, Louisiana State University Museum of Natural Science; WAM, W.A. Matamoros; USM, University of Southern Mississippi; STRI; Smithsonian Tropical Research Institute.

	Tissue No.	Voucher No.	Country	Drainage	GenBank
1	WAM 08–1264	USM-15303	Honduras	Río Coco	KY488518
2	LSUMZ-F 2944	LSUMZ 15444	Nicaragua	Río San Juan	KY488541
3	STRI	-	Panama	Río Indio	KY488522
4	WAM 07–346	USM-34086	Honduras	Río Aguan	KY488532
5	LSUMZ-F 2600	LSUMZ 15303	Nicaragua	Río Prinzapolka	KY488529
6	LSUMZ-F 3311	LSUMZ 15630	Honduras	Río Motagua	KY488526
7	LSUMZ-F 3309	LSUMZ 15630	Honduras	Río Motagua	KY488523
8	LSUMZ-F 3544	LSUMZ 15965	Honduras	Laguna Guiamoreto	KY488520
9	WAM 06–175	USM-31757	Honduras	Río Cuero y Salado	KY488517
10	-	-	Honduras	Río Lancetilla	HM193454
11	LSUMZ-F 1731	LSUMZ 14779	Costa Rica	Río Sixaola	KY488540
12	STRI -	-	Panama	Río Miguel de la Borda	KY588538
13	WAM 08–2560	USM-43170	Honduras	Río Cuero y Salado	KY488531
14	LSUMZ-F 3696	LSUMZ 15667	Honduras	Laguna El Cacao	KY488528
15	STRI -	-	Panama	Río Miguel de la Borda	KY488525
16	STRI -	-	Panama	Río Chagres	KY488537
17	LSUMZ-F 2775	LSUMZ 15372	Nicaragua	Río Wawa	KY488519
18	WAM 06–133	USM-31728	Honduras	Río Papaloteca	KY488516
19	STRI -	-	Panama	Río Grande	KY488542
20	LSUMZ-F 3697	LSUMZ 15667	Honduras	Laguna El Cacao	KY488539
21	LSUMZ-F 2828	LSUMZ 15425	Nicaragua	Río Wawa	KY488536
22	LSUMZ-F 2599	LSUMZ 15303	Nicaragua	Río Prinzapolka	KY488534
23	WAM 08–1265	USM-44989	Honduras	Río Coco	KY488533
24	LSUMZ-F 2601	LSUMZ 15303	Nicaragua	Río Prinzapolka	KY488535
25	WAM 07–345	USM-34086	Honduras	Río Aguan	KY488530
26	LSUMZ-F 3698	LSUMZ 15667	Honduras	Laguna El Cacao	KY488527
27	LSUMZ-F 3310	LSUMZ 15630	Honduras	Río Motagua	KY488524
28	LSUMZ-F 3545	LSUMZ 15965	Honduras	Laguna Guiamoreto	KY488521
29	-	-	Belize	Monkey River	GU736991
30	LSUMZ-F 5590	LSUMZ 16458	Guatemala	Lago Izabal	KY488513
31	LSUMZ-F 5591	LSUMZ 16458	Guatemala	Lago Izabal	KY488514
32	LSUMZ-F 5589	LSUMZ 16458	Guatemala	Lago Izabal	KY488515
33	-	-	Nicaragua	Río Escondido	KU854721
34	-	-	Belize	Stann Creek	KU854719
35	-	-	Belize	Stann Creek	KU854720

### Ethics statement

All collecting was conducted under appropriate scientific, collection, museum, and export permits for Guatemala (Consejo Nacional de Áreas Protegidas permit 00088–2013), Honduras (Instituto Nacional de Conservación y Desarrollo Forestal, Áreas Protegidas, y Vida Silvestre permit DVS-ICF-03302009), Nicaragua (Ministerio del Ambiente y Recursos Naturales permit DGPN/DB/DAP-IC-0008-2010), Costa Rica (Museo de Zoología, Universidad de Costa Rica), and Panama (Autoridad Nacional del Ambiente permit SC/A-17-11). Work and sampling procedures were conducted under IACUC approval 09–002 at Louisiana State University.

### Molecular phylogeography

Whole genomic DNA was extracted from tissues using the Qiagen DNeasy Tissue Kit (Qiagen Inc., Valencia, CA). The mitochondrial cytochrome *b* gene (cyt *b*) has often been useful for assessing phylogeographic patterns within fishes, given its high level of variability and ease of amplification and sequencing. While deep interpretations based solely on this marker are limited, there is utility to assessing general patterns with this gene (e.g. fast mutation rate, previous sequences available; [9,10,20]). The cyt *b* gene was amplified using primers GluDG.L and H6C6B6, as well as with PCR protocols from [21]. PCR products were visualized on a 0.8% agarose gel and compared to a standard for assessment of presence, size, and intensity of amplified fragments. PCR products were sequenced at Beckman Coulter Genomics Facility (Danvers, MA). Chromatographs were checked by eye for ambiguities and manually aligned using Geneious [22]. Sequences were submitted to GenBank and accession numbers for sequences are reported in Table 1.

Phylogenetic trees were inferred using Parsimony and Bayesian Inference methodologies in the programs PAUP* [23] and Mr. Bayes v.3.2.6 [24], respectively. Individuals of *V*. *melanura*, sister species to *V*. *maculicada*, were used as outgroups (GenBank accession numbers AY843418, KU854724, KU854725, KU854728). For the parsimony analysis, all characters were equally weighted, and a heuristic search was performed with unordered and unweighted data. Tree Bisection Reconnection (TBR) branch swapping was executed for 1000 random step-wise additions, and a bootstrap analysis was performed for 100 pseudoreplicates. The model of evolution employed for Bayesian tree inference was selected using the Akaike Information Criterion (AIC) in jModelTest [25–26]. Four independent runs were performed with 7 million generations each, with trees retained every 100 generations. Stationarity was assessed using Tracer 1.5 [27]. The first 10% of trees were discarded as burn-in, and a 50% Majority Rule consensus tree was generated for each phylogenetic analysis. Average sequence divergence was calculated as uncorrected p-distances using Mega v. 5 [28]. TCS xxx [29] was used to examine relationships among *V*. *maculicauda* cyt *b* haplotypes using statistical parsimony [30]. An unrooted haplotype network was generated using a 95% connection limit. Signals of historical population growth were tested by estimating Tajima’s *D* [31] and Fu’s *F* [32] in Arlequin [33] against a null hypothesis of neutrality and constant size. Recent population expansion is one of several factors or processes that can lead to negative values for these statistics. Additionally, mismatch distribution was used to test the null hypothesis of spatial expansion in Arlequin [33]. Harpending’s Raggedness index was calculated to test the fit between expected and observed results and significance was assessed with 1000 permutations.

### Niche modeling and paleodistribution

Ecological niche models (ENMs) were used to test for changes in the distribution of *V*. *maculicauda* through time. The occurrence dataset was generated based on museum specimen records of *V*. *maculicauda*. All records of the species were compiled from FishNet2 (www.fishnet2.net). A small number of records possessed associated latitude and longitude coordinates, thus latitude and longitude records were determined for all specimens using the online georeferencing platform GeoLocate (www.museum.tulane.edu/geolocate/). All georeferenced localities were visually examined to ensure accuracy (S1 Table).

The ENMs were constructed using Maxent v.3.3.3k [34], which overlays georeferenced presence data onto a set of environmental layers. The software then characterizes what environmental conditions are best correlated with given organism. Thus, the spatial output from MaxEnt estimated the fundamental niche for *V*. *maculicauda*.

Climate layers used in the models were downloaded from WorldClim (http://www.worldclim.org/bioclim). The total 19 climate layers were each clipped down to our area of interest in Central America. Clipping the layers reduced the amount of pseudoabsences detected by Maxent due to presence-only data [35]. In addition to the 19 Bioclim layers, we used an altitude layer that was included with MaxEnt. We tested for correlations among climate variables using the SDM toolbox (http://sdmtoolbox.org/) in ArcGIS to produce a covariance matrix. If two layers were correlated, we retained the climate layer that appeared most biologically meaningful. In total, 12 climatic variables were used in our analyses: Bio2 = Mean Diurnal Range (temperature), Bio3 = Isothermality, Bio4 = Temperature Seasonality, Bio6 = Min Temperature of Coldest Month, Bio7 = Temperature Annual Range, Bio10 = Mean Temperature of Warmest Quarter, Bio12 = Annual Precipitation, Bio13 = Precipitation of Wettest Month, Bio15 = Precipitation Seasonality, Bio17 = Precipitation of Driest Quarter, Bio18 = Precipitation of Warmest Quarter, Bio19 = Precipitation of Coldest Quarter. Altitude was included in our present-day model but was not used for modeling paleodistributions because there were no available layers in the latter.

Paleoclimatic models were generated for three historical time periods: mid-Holocene (6,000 years ago), Last Glacial Maximum (LGM; 21,00 years ago), Last Interglacial (LIG; 140,000–120,000 years ago). Many different general circulation models (GCM) exist which can be used to produce some of these historical climate layers, thus we tested layers from multiple GCMs (Table 2) and used the highest layer resolutions available for each GCM. Results were slightly different among GCMs for LGM and mid-Holocene time periods. The final analyses used layers generated with the MPI-ESM-P model (Max Planck Institute for Meteorology), which focused on both oceanic and freshwater variables [36] and corresponded best with the evolutionary history of this species.

**Table 2:** General circulation models (GCMs) tested during ENM analyses.

Time Period	Source	Resolution
Present	http://www.worldclim.org/current	30s
Last Inter-Glacial	Otto-Bliesner et al. 2006	30s
Last Glacial Maximum	CCSM4	2.5min
	MIROC-ESM	2.5min
	MPI-ESM-P	2.5min
Mid Holocene	CCSM4	30s
	MIROC-ESM	30s
	MPI-ESM-P	30s

Present-day and paleoclimatic models were assembled in ArcMap v.10.3 based on spatial output from MaxEnt. All models were compiled from 15 iterations. Thresholds for determining the fundamental niche were set using Maximum training sensitivity plus specificity values for each iteration in MaxEnt following Newman and Austin [35]. Results were stacked in ArcMap.

## Results

### Molecular phylogeography

**Fig 2:**
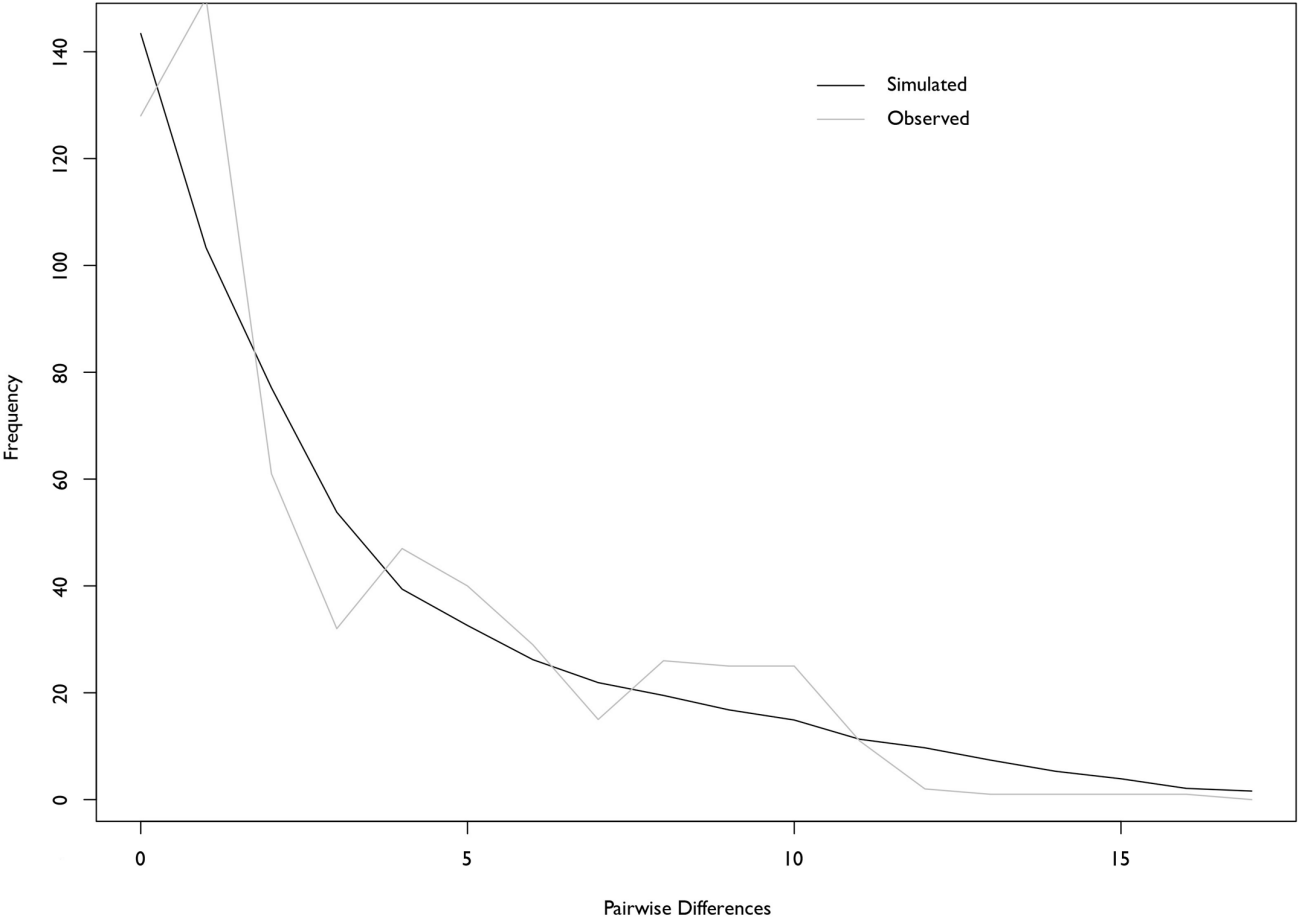
Mismatch distributions of pairwise nucleotide differences. Mismatch distributions showed a unimodal pattern and no significant differences between observed and simulated frequencies.

The cyt *b* alignment was 1137 base pairs in length. Little genetic divergence was observed from samples collected throughout the range of *V*. *maculicauda*. The average sequence divergence was only slightly higher than zero (0.2%) based on uncorrected p-distances. The phylogeny obtained from Bayesian analysis (Fig 1) showed little mitochondrial phylogeographic structure in this widespread cichlid species with low support (posterior probability). However, a slight separation was observed between populations in Belize and Guatemala (north of the Motagua Fault), and areas in more southern portions of Central America. Eleven haplotypes were recovered, with a general star-like network pattern (Fig 1). While only separated by two mutations, there is geographic separation between haplotypes from Belize and Guatemala compared to areas in the southern part of the range. Additionally, population expansion was statistically supported in *V*. *maculicauda* based on a negative Tajima’s *D* (-2.136, P<0.005) and negative Fu’s *F* (-26.185, P<0.005). Mismatch distribution was unimodal, with a low and nonsignificant Harpending’s Raggedness index (Fig 2; 0.0289, P = 0.92).

### Niche modeling and paleodistribution

**Fig 3:**
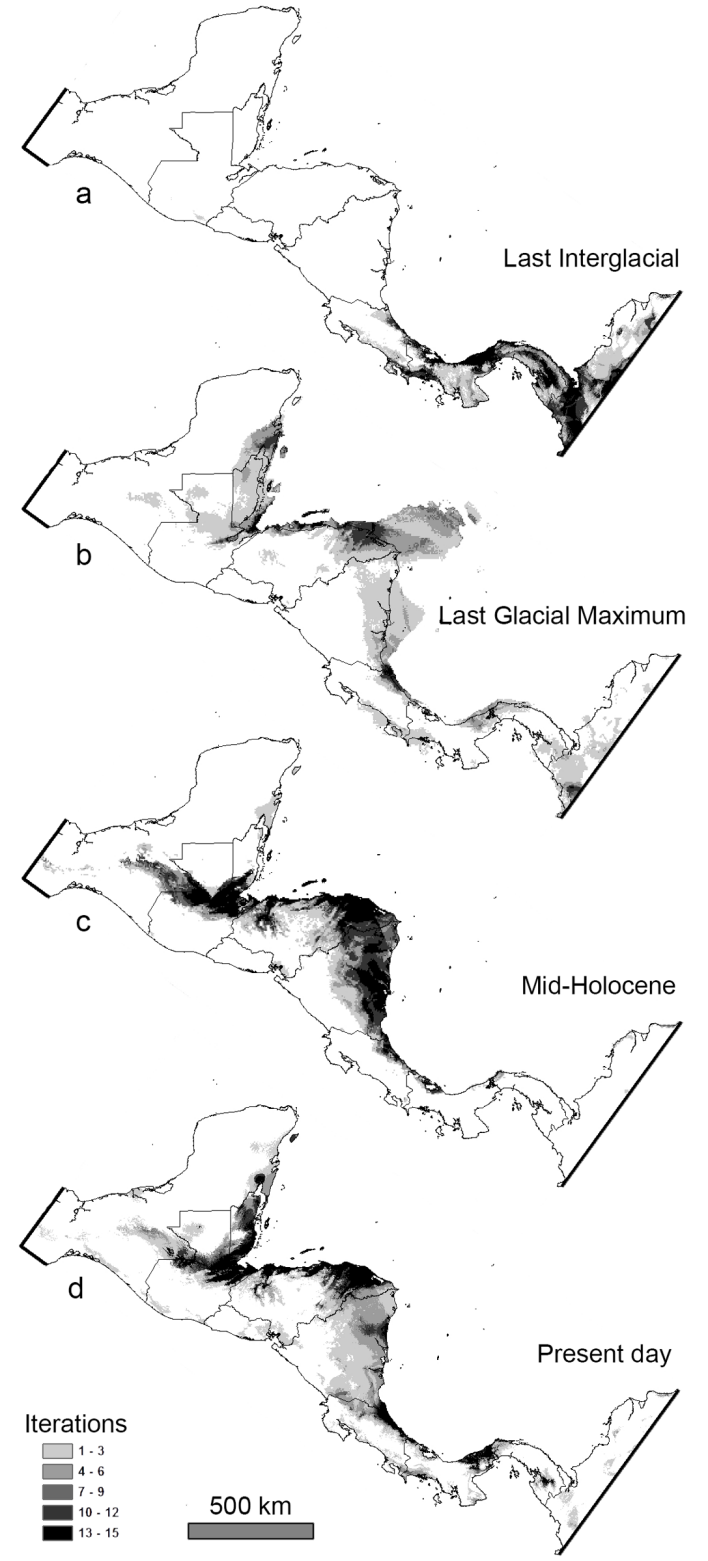
Paleodistribution models (a, Last Interglacial; b, Last Glacial Maximum; c, mid-Holocene) and d, present-day ecological niche model for *Vieja maculicauda*. Darker areas indicate presence in more iterations of the model and therefore the highest probability of occurrence. For the LGM model, extended coastlines are a result of lower sea levels.

**Fig 4:**
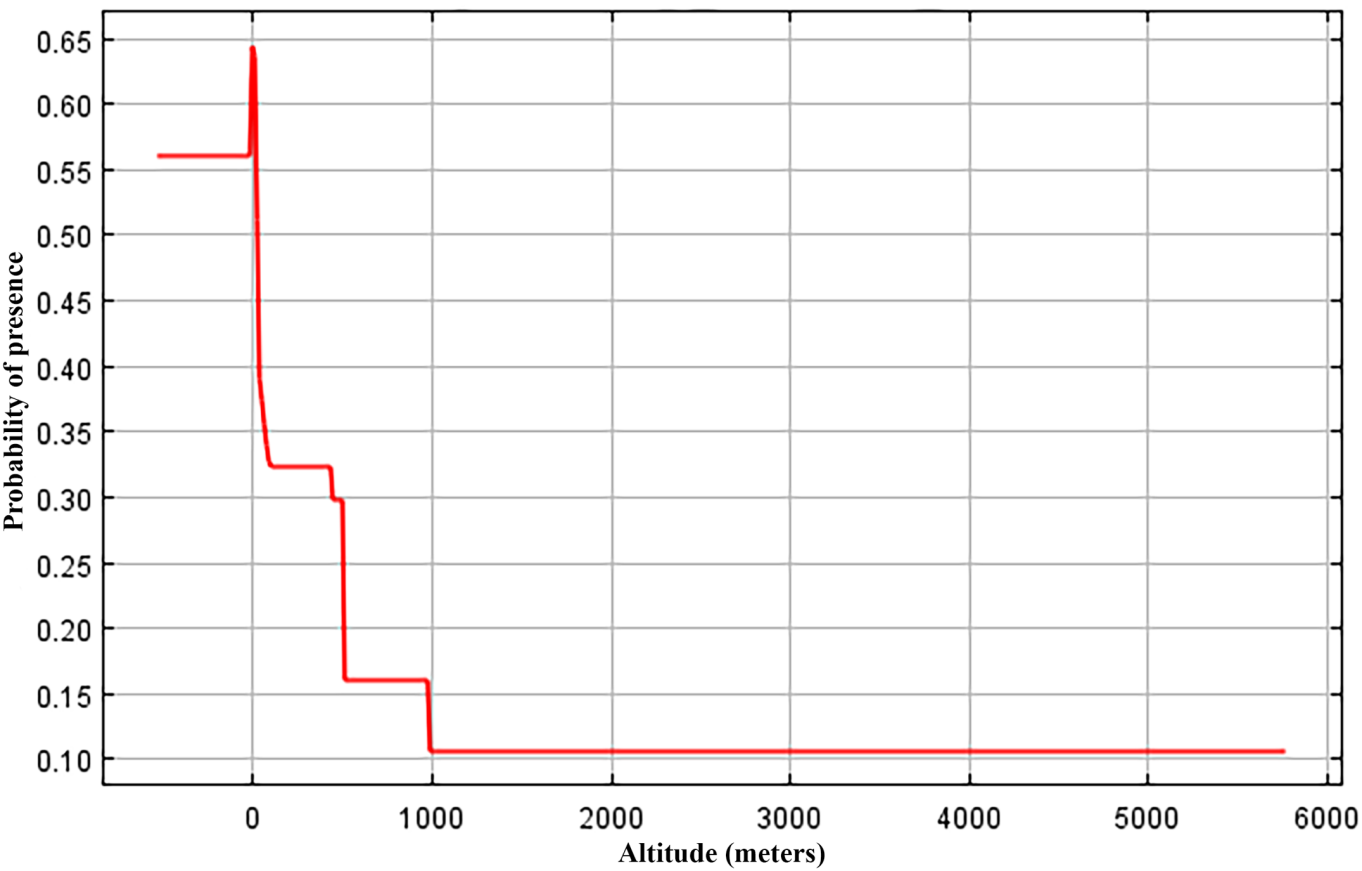
Graph showing probability of presence based on altitude (in meters) generated in ecological niche models for *Vieja maculicauda*.

Overall the present-day ENM generated for *V*. *maculicauda* is congruent with the known distribution of the species (Fig 3), as well as general observations on the lowland habitat preference of this species [11,17,37–38]. Each ENM shows the stacked model output from 15 iterations in MaxEnt. Darker areas indicate regions that appeared more frequently in iterations and therefore show the highest probability of occurrence (Fig 3). Response curves depicted the dependence of predicted habitat suitability as related to individual climatic (i.e. WorldClim) variables based on the generated ENM. *Vieja maculicauda* had high values predicted for habitat suitability (approximately 75%) at or below sea level; however, this percentage decreased drastically (approximately 25%) once elevation reached slightly above sea level (Fig 4).

The LIG model showed the only suitable climatic habitat for this species to be in Lower Central America (Costa Rica and Panama) and northern South America (Fig 3a). All regions north of this area in Central America were not predicted to have been preferable for *V*. *maculicauda* during this time period. The MPI model for the LGM and mid-Holocene were found to be the most biologically relevant (as compared with MIROC and CCSM4 GCMs), and projections for this model are used for subsequent analyses. The LGM model showed suitable climatic habitat in coastal or near-coastal areas along the Caribbean of Central America and Mexico from the Eastern Yucatan and Belize to Nicaragua (Fig 3b). Suitable, but patchy areas were also recovered in Nicaragua, Costa Rica, and Panama. The mid-Holocene model recovered suitable climatic habitat from southern Belize south to Panama, with a break in northwestern Panama (Fig 3c).

## Discussion

*Vieja maculicauda* is the most naturally widespread cichlid in Central America [11]. Results of the present study show slight divergence for this species in Central America for populations north of the Motagua Fault and those south of this area (Fig 1). The low genetic diversity is notable because other freshwater fishes without this lowland restriction exhibit more phylogeographic structure on the same genetic marker [8–9,16,39]. The lack of genetic divergence recovered suggests that the region was recently colonized by *V*. *maculicauda*. *Belonesox belizanus* and *Mayaheros urophthalmus* are two species with similar habitat preferences that show relatively congruent patterns of molecular divergence [40–41].

These results could be attributed to the choice of using a mitochondrial gene; however, prior work illustrates the lack of variation in traditional nuclear markers for phylogeographic resolution of Middle American cichlids [21,42]. Future work aimed at incorporating fine-scale genomic data (e.g. SNPs) might find some phylogeographic structure within this species, and of course, additional specimens may also aid in finding greater genetic diversity. Using genomic data on a larger sampling was beyond the scope of the present study. However, demographically, genetic data indicate population expansion for *V*. *maculicauda* based on the significant and negative Tajima’s *D* and Fu’s *F* values, as well as unimodal distribution and low nonsignificant Harpending’s Raggedness index from mismatch distribution. This has also been illustrated in other Middle American cichlids based on cyt *b* sequences (genus *Amatitlania*, [43])

Niche modeling corroborated distributional evidence from collections that suggested this species is typically found in lowland habitats. Present-day ENM analyses projected onto the geographic range of the species, in addition to the percent occurrence plot with altitude, supports this relatively narrow habitat preference of this species within its wide distribution. While records documenting the occurrence of *V*. *maculicauda* from elevations above sea level do exist (up to around 200m), the results from the ENM support lower elevations as most optimal climatic habitat for the species.

Previous studies have illustrated the utility of ENMs for generating hypotheses to estimate the effect of changes in climate on the paleodistribution of species [2,5,35,44]. We used this tool-kit to test the hypothesis of a directional pattern of northern to southern expansion for this species based on additional evidence strongly supporting a northern Middle American origin of the clade to which it belongs [11,18]. Contemporary ENMs were congruent with the observed present-day distribution of *V*. *maculicauda*. This result suggests that the climatic ENM accurately represents the distribution of this species in Central America. Results suggest a general pattern of northern to southern expansion from LGM to present, supporting our initial hypothesis.

The LIG paleodistribution model shows a lack of suitable habitat in northern Central America 140,000–120,000 years ago. Based on independent phylogenetic evidence [11,18] we do not interpret this ENM result as a hypothesis of complete absence of the species from the northern Central America. In the absence of any evidence to suggest a South American origin for the species we offer two alternative explanations: (1) Either the species originated in the time period between the LIG and LGM, or (2) the distribution in the LIG was quite small or restricted in northern Middle America. The paleodistribution model for the LGM supports the latter possibility. Other cichlid species not generally associated with lowland habitats may show a wider distribution during the LIG [11]. Such comparisons will be an interesting area for future paleodistribution modeling. Additionally, multiple models are available for the LGM and mid-Holocene time periods, but only one for the LIG. Thus, future development of additional LIG GCMs may produce novel hypotheses.

During the LGM, sea levels were lower exposing large areas of land (Fig 3b). The suitable habitat for *V*. *maculicauda* was predominantly in Belize and the lowlands of northeastern Honduras and northwestern Nicaragua, encompassing the present-day region of La Mosquitia, which continues to consist of large expanses of lowland forest and floodplain [45,46]. The cooler and more xeric conditions of the LGM potentially limited the extent of how far and wide *V*. *maculicauda* could disperse; however, this expansion in distribution corroborates studies that have shown an increase in the distribution of tropical species during the LGM [6].

ENMs suggest the greatest range expansion occurred in the time period from the LGM to the mid-Holocene. Contrasting the drier and slightly cooler conditions of the LGM, the mid-Holocene brought more moisture and warmer conditions [2]. Changing climatic conditions likely allowed *V*. *maculicauda* to expand its range considerably throughout more southern portions of Central America (southern Nicaragua, Costa Rica, and Panama). The southern-most area identified as suitable habitat in the mid-Holocene was in the vicinity of the Río Chagres in Panama, which is congruent with the southern-most limit of the species’ contemporary distribution. Additionally, this range expansion is supported by the negative Tajima’s *D* and Fu’s *F* values estimated from mitochondrial sequence data.

ENMs estimate the fundamental niche of a species and almost certainly differ in some regions from the realized niche. The contemporary ENM, as well as projected paleodistribution maps for the LGM and mid-Holocene, identify suitable climatic habitat in some portions of Central America where *V*. *maculicauda* does not occur. For instance, various areas in the greater Río Usumacinta basin in Mexico and northern Guatemala were recovered as suitable habitat. *Vieja maculicauda* does not occur in the Río Usumacinta itself; however, its sister species and other relatives are endemics or natives of the large drainage basin and rivers that share a geological and hydrological history with the Río Usumacinta. Additionally, the Bay Islands off the Caribbean coast of Honduras (Roatán, Guanaja) were found to have some of the highest probability of occurrence for *V*. *maculicauda* in the mid-Holocene and present-day; however, the species does not occur on these islands [38]. The marine barrier separating these islands from the mainland is likely the biggest factor restricting this species from occurring there.

This study shows the utility of integrating various sources of information to formulate hypotheses of contemporary and historical distributions of organisms. Results of genetic and ENM analyses suggest *V*. *maculicauda* was narrowly distributed in Northern Middle America during the LIG and expanded its range southward throughout the Caribbean slope of Central America during the LGM and mid-Holocene as temperatures warmed and moisture increased. Compared to its sister species *V*. *melanura* or other closely related species, *V*. *maculicauda* has a vastly greater geographic distribution. While expansion during the LGM is supported in various tropical species [6], closely related cichlids did not respond to the same degree as *V*. *maculicauda*. As temperatures and sea levels continue to rise in the future, expansion or contraction in the distribution of this species seems probable.

## Supporting information

S1 Table Geographic coordinate data. Latitude and longitude data for collection localities for *Vieja maculicauda*.
(XLS)
